# [^18^F]fluoro-ethylcholine-PET Plus 4D-CT (FEC-PET-CT): A Break-Through Tool to Localize the “Negative” Parathyroid Adenoma. One Year Follow Up Results Involving 170 Patients

**DOI:** 10.3390/jcm10081648

**Published:** 2021-04-13

**Authors:** Constantin Smaxwil, Philip Aschoff, Gerald Reischl, Mirjam Busch, Joachim Wagner, Julia Altmeier, Oswald Ploner, Andreas Zielke

**Affiliations:** 1 Department of Endocrine Surgery, Endokrines Zentrum Stuttgart, Diakonie-Klinikum Stuttgart, 70176 Stuttgart, Germany; buschm@diak-stuttgart.de (M.B.); Wagner@diak-stuttgart.de (J.W.); julia.altmeier@diak-stuttgart.de (J.A.); andreas.zielke@diak-stuttgart.de (A.Z.); 2 Department of Nuclear Medicine and PET-CT Centre, Institute of Diagnostic and Interventional Radiology, Diakonie-Klinikum Stuttgart, 70176 Stuttgart, Germany; aschoff@diak-stuttgart.de; 3 Department of Preclinical Imaging and Radiopharmacy, Werner Siemens Imaging Center, Eberhard Karls University of Tuebingen, 72076 Tuebingen, Germany; Gerald.Reischl@med.uni-tuebingen.de; 4 Cluster of Excellence iFIT (EXC 2180) Image Guided and Functionally Instructed Tumor Therapies, University of Tuebingen, 72076 Tuebingen, Germany; 5 Department of Internal Medicine, Endocrinology, Endokrines Zentrum Stuttgart, Diakonie-Klinikum Stuttgart, 70176 Stuttgart, Germany; Ploner@diak-stuttgart.de

**Keywords:** parathyroid adenoma, hyperparathyroidism, PET-CT, FEC, FCH

## Abstract

Background: The diagnostic performance of [^18^F]fluoro-ethylcholine-PET-CT&4D-CT (FEC-PET&4D-CT) to identify parathyroid adenomas (PA) was analyzed when ultrasound (US) or MIBI-Scan (MS) failed to localize. Postsurgical one year follow-up data are presented. Methods: Patients in whom US and MS delivered either incongruent or entirely negative findings were subjected to FEC-PET&4D-CT and cases from July 2017 to June 2020 were analyzed, retrospectively. Cervical exploration with intraoperative PTH-monitoring (IO-PTH) was performed. Imaging results were correlated to intraoperative findings, and short term and one year postoperative follow-up data. Results: From July 2017 to June 2020 in 171 FEC-PET&4D-CTs 159 (92.9%) PAs were suggested. 147 patients already had surgery, FEC-PET&4D-CT accurately localized in 141; false neg. 4, false pos. 2, global sensitivity 0.97; accuracy 0.96, PPV 0.99. All of the 117 patients that already have completed their 12-month postoperative follow up had normal biochemical parameter, i.e., no signs of persisting disease. However, two cases may have a potential for recurrent disease, for a cure rate of at least 98.3%. Conclusion: FEC-PET&4D-CT shows unprecedented results regarding the accuracy localizing PAs. The one-year-follow-up data demonstrate a high cure rate. We, therefore, suggest FEC-PET-CT as the relevant diagnostic tool for the localization of PAs when US fails to localize PA, especially after previous surgery to the neck.

## 1. Introduction

Primary hyperparathyroidism (pHPT) is the third most common endocrine disorder, with the highest incidence in postmenopausal women. Asymptomatic disease is common and severe disease with renal stones and metabolic bone disease arises less frequently now than it did 20–30 years ago [1]. pHPT is diagnosed by elevated serum calcium and parathyroid hormone (PTH) levels. In about 90% of cases, it is caused by a single parathyroid adenoma (PA) and less often by parathyroid hyperplasia due to multiglandular disease. pHPT can be cured only by surgical removal of the parathyroid adenoma (PA) [2]. Because most patients will only have one PA, they benefit from focused interventions. The success of such “targeted” parathyroid surgery relies on a sensitive and accurate preoperative imaging technique—and an experienced surgeon. The results of focused operations of preoperatively unequivocally localized PA are largely equivalent to a formal, bilateral neck exploration [3]. PAs can be localized by multiple modalities such as high-resolution (7.0–10.0 MHz) ultrasonography (US), computed tomography (CT), magnetic resonance imaging (MRI), and scintigraphy. Parathyroid scintigraphy involves a number of different radiotracers and protocols but [99mTc]Tc-Sestamibi (MS) is the most often used. However, up to one-third of patients with adenomas will be negative with either US or MS [4].

During the last two decades, the role of positron emission tomography/computed tomography (PET/CT) imaging using specific PET tracers has been highlighted. Several PET radiotracers have been introduced for the assessment of pHPT. One of the more commonly used is [^11^C]methionine. Recently, the feasibility of [^18^F]fluorocholine (FCH) and [^18^F]fluoro-ethylcholine (FEC) to localize PAs has been initially reported in case reports and small case-series, but its broader application was recently presented in a clinical study involving 139 operated patients [5]. Most of these reports focused primarily on the degree of agreement between preoperative imaging and intraoperative findings and highlighted the diagnostic parameter. However, most of these studies have not present long term cure rates, hence the clinical utility of FEC/FCH-PET-CT as a diagnostic tool for the localization of PA has yet to be determined.

Here, we present data of a consecutive series of patients who had [^18^F]fluoro-ethylcholine-PET-CT plus 4D-CT (FEC-PET&4D-CT) to localize PA(s) in biochemically proven pHPT when either ultrasound (US) or MIBI-Scan (MS) failed to localize. Moreover, and for the first time, postsurgical one-year follow up data are presented allowing for an assessment of the clinical utility of this new imaging tool.

## 2. Materials and Methods

### 2.1. Study Population

Between July 2017 and June 2020, FEC-PET&4D-CT was employed in patients with biochemically proven pHPT in whom US and MS delivered either incongruent or entirely negative findings. Patients that had concordant results during US and MIBI and, thus, double localized PAs were not included into this study. Irrespective of the findings of any of the imaging tools, all patients were offered cervical explorations with intraoperative PTH-monitoring (IO-PTH) and enrolled into our centers structured postoperative follow up program, to determine the short as well as long term results of cervical exploration.

### 2.2. Preparation of [^18^F]Fluoro-Ethylcholine (FEC)

For synthesis of FEC for human application, the method of Hara et al. [6] was improved and adopted to a TRACERlab FX_F-N_ automated system (GE Healthcare, Münster, Germany) in a certified GMP environment. Briefly, [^18^F]fluoride was produced at a PETtrace cyclotron (GE Healthcare, Uppsala, Sweden) and trapped on an anion exchange cartridge (Waters, Milford, MA, USA). Subsequently, radioactivity was eluted and azeotropically dried in presence of Kryptofix 2.2.2 (Merck, Darmstadt, Germany). In a first step, labelling of 1.2-bis(tosyloxy)ethane (Aldrich, Taufkirchen, Germany) with [^18^F]fluoride/[K/2.2.2] in acetonitrile yielded 2-[^18^F]fluoro-ethyltosylate, which was purified by HPLC and reversed phase solid phase extraction (RP SPE, Waters, Milford, MA, USA). In a second step, by reaction of the labelled tosylate with *N*,*N*-dimethylaminoethanol (DMAE) and subsequent cation exchange SPE (Waters, Milford, MA, USA) purification, FEC was obtained within 55 min, overall. After sterile filtration product volume was ca. 10 mL in buffered saline. Radiopharmaceutical quality control was performed following European Pharmacopoeia (Ph. Eur.) rules. Purity, pH, endotoxin content and sterility met the requirements for parenteralia.

### 2.3. PET/CT Technique

PET/CT examinations were performed on a Discovery 600—PET/CT-Scanner (GE Healthcare, Milwaukee, WIS, USA). One hour after intravenous administration of 200 MBq of FEC. A nonenhanced CT scan preceded the PET acquisition and was used for attenuation correction. The PET acquisition covered the neck and the chest from the angle of the mandible to the diaphragm (static acquisition, 2 to 3 bed positions, 4 min per bed position) [7]. Subsequently the contrast enhanced CT scans were acquired, comprising a scan in arterial phase with bolus tracking technique and a scan in venous phase with a delay of 80 s [8]. CT and PET images were reconstructed using iterative algorithms (ASiR and SharpIR respectively). For quantitative PET assessment, SUVmax of lesions was recorded.

### 2.4. Parathyroidectomy

Patients underwent guided, focused minimal invasive parathyroidectomy with intraoperative neuromonitoring and intraoperative PTH-assay (IO-I-PTH, future diagnostics, 6603 BN Wijchen, The Netherlands) following standards set forth by current guidelines [9,10]. In the case of multiglandular disease, a standardized bilateral exploration of the neck was performed.

All individuals included in this series underwent surgery at a high-volume center of reference for thyroid and parathyroid surgery, certified by the German Association of Surgery (DGAV) [11]. All operations were done by one of 5 certified endocrine surgeons, using standardized techniques with a focus to apply minimal invasive techniques whenever possible. These techniques do not mandate extended dissection to visualize all four parathyroid glands if the enlarged gland is readily exposed and intraoperative PTH testing is indicative of biochemical cure.

### 2.5. Postsurgical Follow up Data

All patients in this series were subject to a standardized perioperative protocol, ensuring biochemical testing for fasting (total serum) calcium and PTH levels between 7 and 8 a.m. on post-operative day (POD) 1 and POD2 as well as POD3 in the rare event, a patient had not yet been discharged. After discharge from the hospital patients were subject to a detailed follow-up program. All patients were followed for at least 12 months postoperatively. To this end, the Endocrine Centers Outcomes Research Unit used structured telephone interviews to assess current medication, laboratory parameter and symptoms of hypo- or hypercalcemia. For the purpose of this analysis, all imaging results (US, MS, FEC-PET-CT, 4D-CT) were correlated to intraoperative findings, histopathology, short term biochemical outcome (i.e., until discharge) as well as the biochemical follow-up data after 12 months. 

To accurately determine the post-surgical cure rate of pHPT it is paramount to obtain biochemical parameter 12 months postoperatively. Historically, the terminology of persisting pHPT has been used for cases in whom biochemical cure is registered only for brief periods, e.g., less than 12–24 weeks, and recurrent pHPT is diagnosed in individuals in whom calcium and PTH have remained within the normal range for periods of more than 3 but less than 12 months [12,13,14,15,16,17]. Hence, in this study biochemical parameter were addressed at 12 months post-surgery to determine the cure rate. In accordance with guidelines, entirely normal values of total serum calcium and PTH were registered as “cure”, whereas elevated total serum calcium above the upper limit of norm concurred by elevated levels of PTH above the upper limit of norm were defined biochemical signs of persisting pHPT. Likewise, simultaneously elevated levels of calcium and PTH, albeit within the normal range, were defined “potential recurrence” and, thus, potential failure.

### 2.6. Handling of Data and Analysis; Definition of Diagnostic Accuracy

All data were prospectively documented as individual perioperative datasets using the hyperparathyroidism-module of the StuDoQ-quality assurance registry of the German Surgical Association (DGAV) [11]. All data computed for this publication were pseudonymized or aggregate non-individual data. There were no missing values. Descriptive statistic was used to summarize patients’ characteristics retrospectively. Continuous variables are reported as mean and standard deviation (SD) including 95% confidence interval and are compared between groups using two-sample independent t-tests. Categorical variables are summarized as frequencies (%). Results of the localization of a PA by FEC-PET-CTs were recorded as follows: surgical removal of a PA at the site predicted by FEC-PET-CT followed by an adequate drop of intraoperative PTH level as well as histopathological confirmation of the PA (i.e., confirmed PA) was recorded as a true positive result. Likewise, surgical removal of a confirmed PA at a site different from the one predicted by FEC-PET-CT was recorded as a false positive result and detection of a confirmed PA in a case where FEC-PET-CT had been non-diagnostic, was recorded as a false negative result.

### 2.7. Ethics Approval and Consent to Participate

This study was approved by the Institutional Review Board of the Diakonie-Klinikum Stuttgart and conducted in cooperation with the Endocrine Centers certified Outcomes Research Unit. All methods were carried out in accordance with the Declaration of Helsinki and the approved guidelines. The data enrolled in this study were obtained from the centers pseudonymized quality assurance database (StuDoQ, a quality assurance database for certified centers of Endocrine Surgery of the German Association of Surgeons, DGAV [11]). Ethical committees determined that the data presented in this article do not represent a human participant research study, do not include personal identifying information, and were carried out for the reason of quality monitoring and assurance. Therefore, this analysis did not require informed consent other than the consent obtained prior to entering data into the StuDoQ Database. However, prior to entering data into the StuDoQ database, all patients gave informed consent, both with regard to FEC-imaging as well as disease specific data acquisition and secondary analysis into the StuDoQ Registry.

## 3. Results

### 3.1. Patient Characteristics

From July 2017 to June 02020, 454 patients were operated for pHPT at our department (64 with prior surgery to the neck (14.1%) including 18 redo-for-HPT (4%)). During this period 171 FEC-PET-CTs were performed. Of these, 152 were performed in combination with 4D-CT (88.9%), in 19 patients no contrast enhanced 4D-CT was performed due to hyperthyroidism or renal dysfunction. Most of the patients had FEC-PET-CT&4D-CT either because of incongruent (48.0%) or entirely negative imaging results (43.3%) by US and/or MS. Nine patients (5.2%) had a negative US without MS performed, and six patients (4.2%) were positive on US and MS but still received FEC-PET-CTs with 4D-CT to confirm the localization because of extensive previous neck surgery. A total of 137 of the 171 patients (80.1%) were female, and 44 were redo-cases (25.8%) either after previous thyroid (*n* = 27, 15.8%) or parathyroid surgery (*n* = 17, 10.0%). All patients had preoperative clearly elevated Calcium (mean 2.78 ± 0.17 mmol/L (2.76–2.81; 95% confidence interval, CI) and elevated PTH (124.6 ± 66.7 ng/L (CI 114.6–134.6). Noticeable was a significant (*p* = 0.02) higher PTH-level and even more significant (*p* = 0.001) higher Calcium-level in patients with previous parathyroid surgery as compared to patients without previous surgery (Table 1).

### 3.2. Localization of PA by Ultrasound, MIBI-Scintigraphy and FEC-PET-CT plus 4D-CT

Overall, FEC-PET-CT with 4D-CT resulted in findings suggestive of a potential PA in 159 patients (93.0%). Of these 171 patients, preoperative diagnostic work up recorded 109 (63.7%) individuals to have been nondiagnostic (negative) on cervical US and 74 (43.3%) were also MS negative; these individuals were thus “double negative” for both US and MIBI. Of the 109 US negative patients FEC-PET&4D-CT suggested a PA in 98 (89.9%) and of the 62 US positive patients in 61 (98.4%) patients.

Of the 74 patients in whom US and MS did not offer a localization of a PA (double negative) FEC-PET&4D-CT suggested a PA in 66 (89.2%) patients.

A total of 26 patients had a positive finding during MIBI-scintigraphy (MS) but were negative on US and a further 9 cases that were negative on US had not undergone MS. Of these 35 patients FEC-PET&4D-CT suggested a PA in 29 (82.9%) patients.

There were 44 patients with previous surgery to either the thyroid or the parathyroid glands. Twelve out of these 44 patients with congruent findings during US and MS still received FEC-PET&4D-CT—mainly because of the extent of preoperative surgery or to rule out parathyreomatosis. In this subset of 44 patients requiring secondary surgery, localization of PA was feasible in all but one, for a detection rate of 97.7% (Table 2).

**Figure 1:**
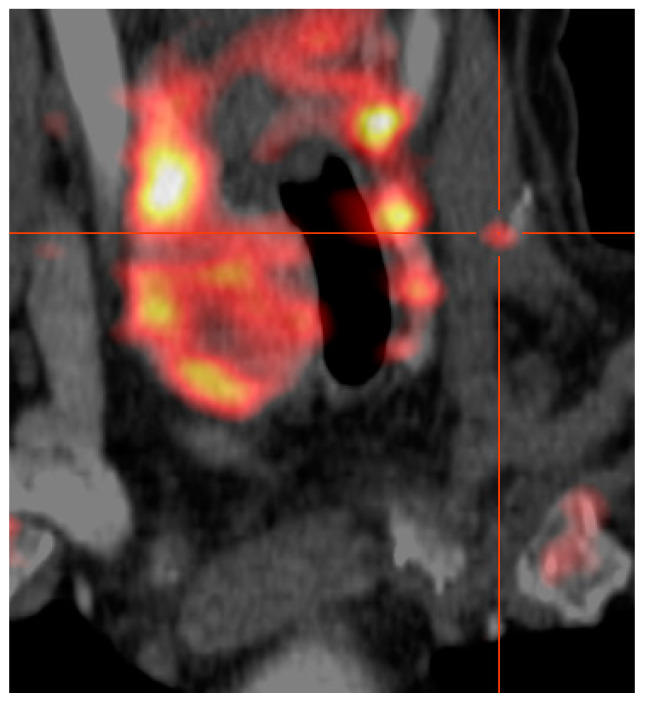
Autotransplanted tissue of a parathyroid adenoma, 18 years after initial surgery depicted by FEC-PET-CT-scan and localized into the left sternocleidoid muscle next to a clip. To the right of the trachea, the right thyroid lobe is seen and to the left of the trachea a chain of parathyroid adenomata, caused by parathyreomatosis is noted.

**Figure 2:**
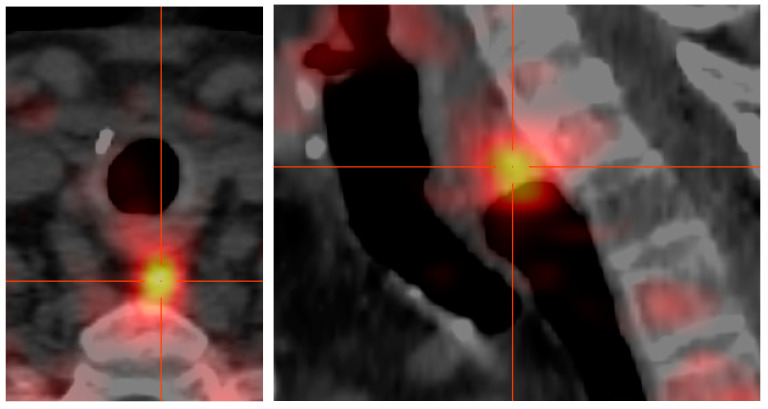
a+b Parathyroid adenoma (PA) of the descended upper left gland in a patient who had total thyroidectomy and central lymph node dissection because of thyroid cancer 10 years earlier.

An example of the imaging resolution of FEC-PET-CT is given in Figure 1, of a patient with recurrent pHPT 18 years after initial surgery for pHPT. The reason for recurrence was a parathyroid spillage parathyreomatosis caused by the primary operation on the left side of the neck. However, FEC-PET-CT demonstrated a discernible FEC-retention next to a clip in the sternocleidoid muscle, revealing a tiny amount of autotransplanted parathyroid tissue (Figure 1). Another example is given in Figure 2 of a patient who had total thyroidectomy and local lymphnode clearance because of thyroid cancer and was diagnosed to have pHPT. This patient had been “double negative” on US and MS during preoperative workup. The prima facie demonstration of the left upper descended parathyroid gland made it possible to choose a focused lateral approach to remove the PA. Because of severe pre-existing conditions (ASA IV: acute on chronic renal failure, severe arterial hypertension, exacerbated COPD, and chronic depression) and because of the pin-pointed localization the procedure was done under local anesthesia in this patient.

### 3.3. Degree of Intraoperative Agreement and Postoperative Long-Term Results

147 of the 171 patients of this cohort already had surgery, including 25 with previous thyroid and 14 with previous parathyroid procedures. The patient’s characteristics of this subset of patients showed no difference to the entire group of patients. Similar to the entire cohort, levels of calcium and PTH were higher in patients with previous parathyroid surgery as compared to patients after thyroid or without previous surgery to the neck.

Of all operated patients FEC-PET-CT plus 4D-CT accurately localized PA(s) to the respective side of the neck in 141 patients; it was found to have been false negative in four and false positive in two patients, respectively, for a global sensitivity of 0.97; accuracy of 0.96 and PPV of 0.99.

FEC-PET-CT successfully localized the PA in 140 of 147 patients, and in all of the patients that had previous surgery to the neck it correctly predicted the site of the PA. We found neither significant difference in SUV nor the size of the PA in the FEC-PET-CT and no difference in the postoperative PTH-decrease for all patients compared to patients after previous surgery. Pathological findings of parathyreomatosis were only demonstrated in the group with previous parathyroid surgery (Table 3).

With regard to the side effects of surgery two patients were treated for postoperative bleeding—one patient had undergone synchronous thyroidectomy for Graves’ Disease and one was a reoperative case for persisting pHPT. There were two cases of temporary vocal cord dysfunction. One patient with previous thyroid resection had a vocal cord paresis contralateral to the current operated side. Another patient had a paresis after a combined procedure involving a simultaneous thyroid lobectomy. Both patients had full recovery of their vocal cord function within six months after surgery, respectively.

The rate of persisting HPT in this series was zero since none of the 117 patients that already have completed their 12-month postoperative follow up had biochemical signs of persistent disease. However, two cases (one case of recurrent pHPT who had repeat surgery for PA 18 years after the primary procedure and one case of pHPT with removal of a solitary PA) had elevated levels of both PTH and Calcium, albeit well within the normal range. Taking account of these two patients, which may have a potential for recurrent disease in the future, the overall cure rate would amount to 115 of 117 cases (98.3%). The cure rate (Table 4) of the subset of double negative patients would amount to at least 49 of 50 (98.0%) and the cure rate of patients with previous surgery to the thyroid and parathyroid to at least 26 of 27 (96.3%).

## 4. Discussion

Historically, bilateral surgical exploration of the neck using surgical protocols that explore all of the possible sites of PA have produced excellent clinical outcome in experienced hands. Over the past two decades there has been a shift towards minimally invasive parathyroidectomy (MIP); a focused operation whereby only the affected PA is removed. Compared to bilateral neck exploration, MIP is associated with smaller incisions, shorter operating time, lower complication rates and faster recovery from postoperative hypoparathyroidism [18,19]. Focused operations, however, rely on preoperative localization of the PA as well as intraoperative PTH-monitoring [9].

There are a number of modalities for preoperative localization such as Ultrasound (US), MIBI-scintigraphy (MS), PET-CT, MRI as well as fine-needle aspiration with measurement of PTH. The most recent guideline (NICE 2019) recommends cervical US and suggests a second modality, such as MS, if it will further guide the surgical approach [20]. Likewise, the American Association of Endocrine Surgeons (AAES) guideline recommends US and another “high-resolution imaging” for operative planning of focused operations [9]. Adherence to these protocols results in favorable clinical outcome: a recent study on 682 patients with pHPT from a tertiary referral center, reported an overall one year cure rate of 97.4% and of 87.5% in the setting of surgery for persisting disease [21].

A recent meta-analysis underscored the utility of US as the first-line tool for the detection of PAs with a pooled sensitivity of 76.1% (95% CI 70.4–81.4%) and positive predictive value (PPV) of 93.2% (90.7–95.3%), respectively [22]. MS had a pooled sensitivity of 78.9% (64–90.6%) and PPV of 90.7% (83.5–96.0%) [23]. In the 20% of cases in whom either or both of these methods fail to localize, PET-CT with [11C]methionine is most often employed. 11C has a half-life of only 20 min, which requires on-site production rendering it unfit for wide-spread use [24]. Using FCH, with the longer-lived 18F (half-life ca. 110 min) the need for an on-site cyclotron is obviated. FCH has only recently been used for the localization of PA in a small number of studies. However, the results have been very encouraging [25].

In this study the rate by which PAs are localized by FEC-PET-CT that had not been detected by US and MS, was found to be well above 90%, and the rate of detection in patients with previous surgery to the neck including reoperations for pHPT was above 90%, too. Our findings are, therefore, consistent with those of other case series [26]. Moreover, and for the first time, this study delivers one year follow up data, confirming a global cure rate for PAs localized by FEC-PET of at least 98.3% and at least 96.3% for PA not localized with US and MS. The clinical utility of FEC-PET-CT is underscored by a cure rate of 100% for patients with previous surgery to the neck including failed parathyroid procedures. Although the number of patients with persisting and recurrent pHPT in this study was only 16, it would still appear, that the use of this novel imaging modality may allow to obtain a control of hypercalcemia more often than has been reported before [14].

Because FCH-PET-CT causes less radiation exposure in comparison to MS [27] and taking account of its impressive diagnostic performance, a recent review already suggested FCH as the one and only “one-stop-shop” imaging modality [24]. US, however, has no radiation exposure and was shown to be a very cost-effective tool (21). Therefore, it is unlikely to be replaced as the first line imaging tool. This is underscored by the results of this current study, where all of the PAs demonstrated by US were confirmed by FEC and—more importantly—also, at the time of surgery. This would suggest the greatest clinical utility of FEC to be found in patients with a negative finding on US.

There are some limitations in this study for critical discussion. As this is a retrospective study, the criteria as to when FEC-PET-CT was performed were not stringent. It is for this reason, that we analyzed the subgroup of cases where the PA was not localized during US and MS. This is a well-defined set of patients in whom additional diagnostic is consented by guidelines, and the results obtained in this subset were comparable to those of the entire cohort.

Another weakness of this study is that some of the patients with FEC-PET-CT have yet to undergo surgery or complete their one year follow up, and, finally, this study did not evaluate the relationship between the success rate of PET-CT and the exact anatomical location of the PA, the size or weight of the specimen removed or the extent of the surgical procedure. At this time, and given the surprisingly small number of PA missed by FEC-PET-CT, we believe, that the analysis of failure of FEC will be feasible as soon as larger data sets from quality assurance registries such as the StuDoQ register become available [11]. Moreover, a protocol for a prospective randomized trial has just been published [28].

Finally, in this cohort of diagnostically challenging patients we used FEC always in combination with 4D-CT, which many consider the current gold-standard diagnostic tool. Because these patients had been preselected for negative US and/or MS, a comparative analysis of the diagnostic effect of either of these tools is therefore not feasible. However, when we decided to add FEC to our escalating protocol of diagnostic procedures, there were no data to expect FEC to stand out with such robust diagnostic performance. Keeping this result in mind, further prospective studies may be warranted, comparing the diagnostic performance of 4DCT and FEC as upfront diagnostic tools.

Despite these limitations, we believe this study has shown that FEC-PET-CT&4D-CT delivers unprecedented results regarding the accuracy by which PAs are localized. This was especially true in clinically challenging scenarios as well as reoperative cases. FEC-PET-CT allowed for focused as opposed to bilateral cervical explorations in almost all of the cases and had a very favorable one-year cure rate.

We therefore consider FEC-PET-CT to be the imaging method of choice for the localization of PAs when US failed to localize the PA, and that it may be particularly helpful to localize PA in patients that already had previous surgery to the thyroid or parathyroid.

## Figures and Tables

**Table 1:** Patients characteristics of 171 FEC-PET-CT&4D-CT from July 2017 to June 2020 for all patients and the two subgroups with previous thyroid and parathyroid surgery. Qualitative data are expressed as numbers; continuous data are expressed as mean ± SD and 95% confidence interval. Reference range Calcium: 2.2–2.6 mmol/L; PTH 12–45 ng/L. * *p* < 0.05 and ^Ɨ^ *p* = 0.001 as compared to patients without previous surgery.

Patient Characteristics			
	All	Prev. Thyroid Proc.	Prev. Parathyroid Proc.
Number (%)	171 (100%)	27 (15.8%)	17 (10.0%)
Sex			
Male	34 (19.9%)	4 (14.8%)	4 (23.5%)
Female	137 (80.1%)	23 (85.2%)	13 (76.5%)
Age (years)	61.5 ± 12.8 (59.5–63.4)	66.1 ± 9.6 (62.5–69.8)	57.0 ± 15.2 (49.7–64.2)
Biochemistry			
Calcium	2.78 ± 0.17 (2.76–2.81)	2.77 ± 0.15 (2.71–2.82)	2.88 ± 0.22 (2.77–2.98) ^Ɨ^
PTH	124.6 ± 66.9 (114.6–134.6)	129.4 ± 56.3 (108.1–150.6)	180.1 ± 115.3 (125.2–234.9) *

**Table 2:** Absolute numbers and percentage of patients with positive and negative localization of a parathyroid adenoma in the preoperative work-up for all patients and subgroups with either previous thyroid or parathyroid surgery and the respective number of positive findings in FEC-PET-CT&4D-CT.

Results of Preoperative Ultrasound (US) and Mibi-Scan (MS) Localization			
	All	Prev. Thyroid pProc.	Prev. Parathyroid Proc.
*n*	171	27	17
US negative	109 (63.7%)	16 (59.3%)	11 (64.7%)
and MS negative	74 (43.3%)	9 (33.3%)	9 (52.9%)
and MS positive	26 (15.2%)	7 (26.0%)	2 (11.8%)
and MS not done	9 (5.3%)	-	-
FEC-Pet-CT&4D-CT pos	94(86.2%)	16 (100%)	10 (90.9%)
US positive	62 (26.3%)	11 (40.7%)	6 (35.3%)
and MS negative	57 (33.3%)	9 (33.3%)	4 (23.5%)
and MS positive	3 (1.8%)	1 (3.7%)	2 (11.8%)
and MS not done	2 (1.2%)	1 (3.7%)	-
FEC-Pet-CT&4D-CT pos	61 (98.4%)	11 (100%)	6 (100%)

**Table 3:** Patients’ characteristics, intraoperative agreement (of preoperative FEC-PET-CT localized parathyroid adenomas) and postoperative results of patients operated after imaging with FEC-PET-CT. Data are presented from the entire cohort of 147 patients and the subgroups with previous thyroid and parathyroid surgery. Qualitative data are expressed as numbers; continuous data as mean ± SD and 95% confidence interval. * One patient with previous thyroid resection developed a vocal cord paresis contralateral to the current operated side and another paresis occurred ipsilateral in a patient with simultaneous hemithyroidectomy. ** One patient had synchronous thyroidectomy for Graves’ Disease, and another had a reoperation for persisting pHPT, respectively (Ref. range Calcium 2.2–2.6 mmol/L; PTH 15–65 ng/L).

Degree of Intraoperative Agreement and Postoperative Results			
	All Operated Patients	Prev. Thyroid Proc.	Prev. Parathyroid Proc.
	147	25	14
Female	119 (81.0%)	22 (88%)	11 (78.6%)
Age (y)	60.8 ± 8.1 (56.8–65.0)	65.4 ± 9.6 (61.6–69.2)	56.9 ± 15.6 (48.7–65.0)
BMI	26.8 ± 3.8 (23.5–30.1)	27.0 ± 5.1 (24.9–29.2)	27.3 ± 3.5 (25.1–29.4)
preop biochemistry			
Calcium (total, mmol/L)	2.79 ± 0.13 (2.72–2.85)	2.78 ± 0.15 (2.72–2.84)	2.92 ± 0.19 (2.82–3.02)
PTH (ng/L)	129.0 ± 58.1 (99.6–158.4)	131.0 ± 58.3 (108.1–153.8)	199.9 ± 117.7 (138.2–231.5)
FEC-PET CT			
pos for PA	140 (95.2%)	25 (100%)	14 (100%)
neg for PA	7 (4.8%)	-	-
size of the PA (mm)	11.0 ± 5.8 (8.1–13.9)	10.8 ± 4.2 (9.1–12.4)	13.7 ± 10.4 (8.3–19.1)
SUV	5.3 ± 1.9 (4.4–6.3)	5.5 ± 2.3 (4.6–6.4)	5.5 ± 2.7 (4.1–6.9)
Histology			
single adenoma	136 (92.5%)	23 (92.0%)	11 (78.6%)
double adenoma	5 (3.4%)	2 (8.0%)	1 (7.1%)
hyperplasia	3 (2.0%)	-	-
parathyreomatosis	2 (1.4%)	-	2 (14.3%)
normal parathyroid gland	1 (0.7%)	-	-
postoperative data			
PTH (ng/L)	26.2 ± 15.1 (18.6–33.9)	24.6 ± 12.9 (19.2–30.0)	31.4 ± 26.9 (16.8–46.1)
Paresis of vocal cord *	2 (1.4%)	1 (4.0%)	-
Postoperative bleeding **	2 (1.4%)	-	1 (7.1%)

**Table 4:** Twelve months biochemical follow up data and cure rate of patients operated after imaging with FEC-PET-CT. Data are presented from the entire cohort of 147 patients enrolled between July 2017 and June 2020 and the subgroups with previous thyroid and parathyroid surgery. Qualitative data are expressed as numbers; continuous data as mean ± SD and 95% confidence interval. Ref. range Calcium 2.2–2.6 mmol/L; PTH 15–65 ng/L) * US and MS negative PAs.

One Year Follow Up Results			
12 Months Follow Up Data	All Operated Patients	Double Neg. *	Prev. Neck Surgery
	117	50	27
PTH (ng/L)	44.3 ± 18.4 (40.6–48.0)	44.5 ± 17.9 (38.9–50.0)	48.9 ± 18.0 (39.5–58.3)
Ca (total, mmol/L)	2.37 ± 0.13 (2.35–2.39)	2.37 ± 0.13 (2.33–2.41)	2.36 ± 0.15 (2.24–2.44)
Paresis of vocal cord	-	-	-
**Cure rate (at least)**	**115 (98.3%)**	**49 (98%)**	**26 (96.3%)**

## Data Availability

Restrictions apply to the data presented in this study. Data were obtained from the StuDoQ Thyroid Quality Assurance Registry of the German Association of Surgeons (DGAVC) and may be available to third parties only upon request to the SAVC/DGAVC and decision at the discretion of the DGAVC.

## Abbreviations

CT	computed tomography
FEC-PET-CT&4D-CT	[^18^F]fluoro-ethylcholine-PET-CT plus 4D-CT
IO-PTH	intraoperative monitoring of parathyroid hormone
MIP	minimally invasive parathyroidectomy
MRI	magnetic resonance imaging
MS	[^99m^Tc]Tc-sestamibi scintigraphy
PA	parathyroid adenoma
PET	positron emission tomography
pHPT	primary hyperparathyroidism
POD	post-operative day
PTH	parathyroid hormone
StuDoQ	Thyroid surgery quality assurance registry of the German Association of Surgeons; StuDoQ|Thyroid
SUV	Standard Uptake Value
US	ultrasound
VC	vocal cord
VCP	vocal cord paresis
